# Student feedback about the use of literature excerpts in Sparshanam, a Medical Humanities module

**DOI:** 10.12688/f1000research.1-49.v1

**Published:** 2012-11-15

**Authors:** P Ravi Shankar, Kundan K Singh, Ajaya Dhakal, Arati Shakya, Rano M Piryani

**Affiliations:** 1 Department of Medical Education, KIST Medical College, Lalitpur, Nepal; 2 Department of Clinical Pharmacology, KIST Medical College, Lalitpur, Nepal; 3 Department of Pediatrics, KIST Medical College, Lalitpur, Nepal; 4 Department of Medicine, KIST Medical College, Lalitpur, Nepal

## Abstract

Medical humanities (MH) modules have been conducted for first year students at KIST Medical College, Lalitpur, Nepal for the last four years. Literature excerpts are widely used in MH programs in developed nations. In Nepal English language literature excerpts had been used previously in two modules. Problems noted were difficulty in comprehending the excerpts and relating them to the Nepalese scenario. The MH module for the 2011 intake was conducted from December 2011 to March 2012. The present study was conducted in the third week of March to obtain student perceptions about use of literature excerpts and suggestions for further improvement using a questionnaire. Literature excerpts used in the module dealt with Nepal and health-related topics. Sixty-eight of the 80 students (85%) participated in the study. The majority were male, self-financing and from urban areas. Respondents felt the excerpts introduced them to different aspects of the medical profession, prepared them for future practice, and underscored the importance of understanding the patients’ feelings. The literature excerpts with which they could identify the most and the least were noted. There were no differences in median enjoyment and effectiveness scores of the literature excerpts according to subgroups of respondents. The suggested benefits of using literature in medical education were similar to those reported previously. Most respondents were able to appreciate the English language excerpts. They felt that Nepali language excerpts and those by Nepali writers could also be included. The findings would be of interest to educators in other developing nations introducing MH modules.

## Introduction

KIST Medical College (KISTMC) is a private medical school in the Lalitpur district of the Kathmandu valley affiliated to the Institute of Medicine, Tribhuvan University for the undergraduate medical (MBBS) course. The college admitted its first batch of students in November 2008 and four batches of students have been admitted since. A medical humanities (MH) module has been conducted for first year medical students right from the first batch ^1^ using small group, activity-based learning methods. Case scenarios, role plays, facilitator presentations and paintings are among the different modalities used to explore various aspects of MH ^2, 3^.

The first author had used literature excerpts during a voluntary module for medical students and faculty members at the Manipal College of Medical Sciences (MCOMS), Pokhara, Nepal ^4^. Literature excerpts were also used during a module conducted for faculty members and medical and dental officers at KISTMC ^5^. Problems were noted with the use of literature excerpts. The excerpts were in English and were mainly obtained from the book ‘Ten years of the medicine and the arts’ ^6^ published by the Association of American Medical Colleges. Participants felt the excerpts did not reflect the Nepalese scenario, the language used was difficult to understand and they could not relate to certain excerpts.

Literature has been widely used in MH programs especially in developed nations. At the Northwestern Medical School in Chicago, United States (US) literature is taught in courses from the first day of medical school through residency ^7^. Literature has been used in the MH program at the University of Missouri-Kansas City School of Medicine in the US ^8^ and during a family medicine clerkship at the University of California, Irvine College of Medicine ^9^. In Asia literature from both Western and Arabic contexts has been widely used at the Weill Cornell Medical College in Qatar ^10^.

Literature has a number of advantages in the education of doctors. It develops skills in observation and interpretation, develops clinical imagination, and promotes clarity of expression ^7^. Literature can foster tolerance for uncertainty inherent in clinical practice, and increase empathy towards patients. A review states that five broad goals are met by the study of literature in medicine. Literary accounts can teach medical students concrete and powerful lessons about the lives of sick people ^11^. Physicians are able to better understand and empathize with narrative stories of patients, literature increases physicians’ expertise in narrative ethics and literary theory can offer new perspectives on the work and genres of medicine.

The MH module at KISTMC is named as ‘Sparshanam’ meaning ‘touch’ in Sanskrit, an ancient South Asian language. The 80 students of the 2011 intake were divided into six groups each consisting of 14 or 15 students. The module was conducted for 90 minutes every Thursday during the early clinical exposure for first year students. Eight sessions were conducted from December 2011 to March 2012. The topics covered during the eight sessions were empathy, what it means to be sick in Nepal, the patient, the doctor, the doctor-patient relationship, the family, the medical student and a wrap up session. Literature excerpts were reintroduced in this module and were used during certain sessions. Keeping in mind previous feedback we used excerpts with relatively simple language and which were directly related to Nepal. The excerpts were by western authors and in the English language. Further information about the excerpts used is provided in the Methods section. The present study was conducted in the third week of March 2012 with the following objectives:

a. Obtain feedback on the literature excerpts used and

b. Get suggestions for further improvement.

## Methods

The study was conducted among first year undergraduate medical (MBBS) students in the third week of March 2012 on conclusion of the Medical Humanities module. The aims and objectives of the study were explained to the students and they were invited to participate. Written informed consent was obtained from all participants. The study was approved by the Institutional Review Board of KIST Medical College.

Literature excerpts were used during certain sessions of the module. Among the books used were ‘The snow leopard’ by Peter Matthiessen ^12^, ‘Three men in a boat’ by Jerome K Jerome ^13^, ‘Window on to Annapurna’ by Joe Stephens ^14^, ‘Aama in America’ by Broughton Coburn ^15^ and ‘The tennis partner’ by Abraham Verghese ^16^. ‘The snow leopard’ is a book about the author’s trek in the remote Dolpo district of Nepal in 1973. He was trekking with a naturalist who was studying mountain goats and the snow leopard. The excerpt used was towards the end of the book where the author was returning back and his Sherpa falls sick. He is treated by a Japanese doctor from a mountaineering expedition. ‘Three men in a boat’ is a humorous account of a boat journey undertaken by three friends on the Thames River. The excerpt deals with the author reading a medical textbook and beginning to imagine that he suffers from the different diseases enumerated. ‘Window on to Annapurna’ is a story of a woman and her husband staying for a year in a Magar village outside the town of Baglung in western Nepal and how they become a part of the life of the villagers. The excerpt used dealt with different faith healing practices which the author had witnessed. ‘Aama in America’ is the story of a Peace Corps worker who stays in a remote village and is adopted by an old Gurung lady as her foster son. Later he takes the old lady to America and shows her his country. The excerpt used describes the lady’s fear that she might die in America away from her family and also describes Gurung funeral rites. ‘The tennis partner’ describes a young Australian student who joins the author’s medical school and who is fighting a losing battle against drug abuse. The excerpts deal with doctors from India emigrating to America and about doctors and drug abuse. The student groups were asked to interpret the excerpt in the context of the topic of the day’s session and present their interpretation to the house using flip charts.

Sixty-eight of the 80 students (85%) participated in the study. Student responses were collected using a questionnaire. Basic information about the respondents like gender, method of financing of medical education, medium of instruction at school, place of family residence and occupation of parents were noted. The questionnaire used is shown below. Free text comments were collected about participants’ perception regarding the use of literature excerpts in Sparshanam, how literature excerpts helped in realizing the objectives of the module, participants’ opinion regarding the use of excerpts mainly by western writers, which of the different excerpts they could identify with the most and the least. Opinion was also sought on what they would suggest to make the exercise more useful, whether they faced difficulties in putting the excerpts in a Nepalese context and if yes, how they overcame these. Similar responses were noted and the frequency of different responses worked out. Common responses were tabulated.

Student feedback questionnaire Student feedback questionnaire about the use of literature excerpts in Sparshanam, the KIST Medical College (Nepal) medical humanities module

Participants were asked to rate on a scale of 1 to 5 their enjoyment of the literature excerpts used and their perception regarding the effectiveness of the excerpts. The mean and median scores were calculated. One sample Kolmogorov-Smirnoff test was used to test the normality of distribution of the scores. Both were found not to be normally distributed and non-parametric tests (Mann Whitney test was used for variables with two subgroups and Kruskal Wallis test for the others) were used to compare the median scores among subgroups of respondents.

## Results

Sixty-eight of the 80 students (85%) participated in the study. Table 1 shows the demographic characteristics of the student respondents. The majority of students were male, self-financing and from urban areas. Only a few students had parents from health related fields.

**Table 1.** Demographic characteristics of student respondents. * The numbers may not add up to 68 and the percentage to 100 in all cases as certain respondents did not complete all demographic details.

**Characteristic**	**Number** (percentage of the total)*
**Gender** Male Female	47 (69.1) 21 (30.9)
**Financing** Scholarship Self-financing	7 (10.3) 60 (88.2)
**Medium of instruction at school** English Vernacular	63 (92.6) 3 (4.4)
**Family residence** Urban Rural	52 (76.5) 12 (17.6)
**Occupation of father** Health related Others	4 (5.9) 63 (92.6)
**Occupation of mother** Health related Others Housewife	3 (4.4) 14 (20.6) 51 (75)

Table 2 shows the respondents’ comments about the use of literature excerpts in the module along with the frequency of the comments. Other interesting comments in addition to those mentioned in the table were “excerpts introduced us to different ways of thinking, and gave us ideas about right and wrong”. Ten students (14.7%) stated they had previous experience with use of literature for educational purposes. Among the books mentioned were ‘Diary of Anne Frank’ and ‘Animal farm’. Regarding how the literature excerpts helped in realizing the objectives of the module, 11 respondents (16.2%) stated it helped by preparing doctors for future practice, 10 (14.7%) said it helped them understand patient’s feelings, eight (11.8%) stated it helped them by highlighting the reality of various issues discussed during the module, while five respondents (7.3%) wrote it helped them in visualizing different situations covered during the MH module.

**Table 2.** Respondents’ comments about the use of literature excerpts in the module.

**Comment**	**Number** **(percentage of total)**
The excerpts introduced us to different aspects of the work done by doctors	16 (23.5)
Introduced problems of society	8 (11.8)
Prepared us for future practice	8 (11.8)
We understood importance of patient’s feelings	8 (11.8)
The excerpts were based on real life situations	8 (11.8)
The excerpts were interesting and informative	7 (10.3)
We learnt about various aspects of the doctor-patient relationship	7 (10.3)
The excerpts were fun	7 (10.3)

Only six respondents (8.8%) were aware of the use of literature in medical education elsewhere. They mainly mentioned the examples of medical schools in western nations without giving specific names. Forty-eight respondents (70.6%) felt the use of literature excerpts by western authors in the module were appropriate. A respondent mentioned, *“Nepal is also rapidly developing. Being in a poor country does not mean we should be away from the modern drama of medical development. And apart from that, we are influenced predominantly by the western world”*. Among the reasons mentioned were the excerpts widened their horizons, they could learn from the western writers, the writers mainly described their experiences in Nepal, they showed cultural aspects of illness, students have been exposed to western literature since childhood, and this type of literature is more easily available. Twelve (17.6%) felt this was not appropriate. Among reasons mentioned were Nepali excerpts were also available, the local context was always better and the excerpts dealt with a different set of problems.

Table 3 shows the literature excerpts with which respondents identified the most along with reasons. A student wrote, *“Three men in a boat, I suppose. Firstly it was an assignment for our group and secondly, when I was younger and just being exposed to medical difficulties, I used to have lots of thoughts about my condition and made certain interpretations which weren’t true”*. Among the books and excerpts with which participants identified the least was ‘Window on to Annapurna’ and ‘Three men in a boat’ (12 respondents each). The reasons mentioned for the first book were difficulty in identifying with the faith healing practices described, difficulty in understanding the excerpts and the respondents not having personal experience of life in less developed areas. For ‘Three men in a boat’ reasons mentioned were the excerpt was difficult to understand and the situation described was not believable. Among the suggestions to make the activity of interpreting literature excerpts more useful were using more literature in Nepali language and written by Nepali authors [14 respondents (20.6%)], students reading the entire book before the session [6 respondents (8.8%)], use of more literature excerpts [5 respondents (7.3%)], and more feedback to be provided by the facilitators and more time for the activity [4 respondents each (5.9%)]. It was also suggested by a respondent that the same literature excerpt could be provided to all the groups.

**Table 3.** Literature excerpts with which respondents identified the most along with reasons.

**Excerpt**	**Number (percentage)**	**Reasons**
**Aama in America** (discussion about Aama’s funeral)	19 (27.9)	Message of love Depicts rural Nepalese society Highlights importance of religion and ceremonies
**The tennis partner** (the resident abusing drugs)	13 (19.1)	Problem all medical students face Risk of the medical profession
**The tennis partner** (emigrating to the United States)	12 (17.6)	This is a common situation Shows doctors are money minded I also plan to emigrate in future
**The snow leopard** (author’s Sherpa getting treated by a foreign expedition doctor)	7 (10.3)	Shows problem of our society Difficulty in getting treatment in rural areas Respondent had personal experience of falling sick while trekking
**Three men in a boat** (author imagining he is suffering from different diseases described in a textbook)	5 (7.3)	The respondents also had personal experience of the same

The median score of respondents with regard to enjoyment of the literature excerpts used and perceived effectiveness of the excerpts used were 4 (maximum score 5). Table 4 shows the median scores according to demographic characteristics of respondents. There were no significant differences in scores according to respondents’ characteristics. Respondents were asked whether they experienced any difficulty in putting the excerpts in the Nepalese context; 40 (58.8%) stated they faced no difficulties while 21 (30.9%) stated they had difficulties. Among the strategies used to overcome the difficulties were group work and consultation, using their imagination and putting themselves in the scenario, and taking help from the facilitators. Fifty-five respondents felt excerpts from works by Nepali authors and in the Nepali language should also be used. A student wrote, *“Yes, it would be a great idea. The modules will be even more interesting and we can relate well. But excerpts from other nations should also be included. This will provide an overall vision on situations”*. They felt excerpts related to medicine and excerpts from rural areas should be used. The excerpts can be obtained from different sources, and a few respondents suggested specific books that can be used while some felt students can themselves supply the excerpts. A large majority [62 respondents (91.2%)] felt literature excerpts should be continued to be used in future modules.

**Table 4.** Median enjoyment and effectiveness scores according to participants’ personal characteristics.

**Characteristic**	**Median** **enjoyment** **scores**	**P value**	**Median** **effectiveness** **scores**	**P value**
**Gender** Male Female	4 4	0.215	4 4	0.303
**Financing** Scholarship Self-financing	4 4	0.697	4 4	0.137
**Medium of instruction** English Nepali	4 4	0.625	4 4	0.852
**Family residence** Urban Rural	4 4	0.660	4 4	0.939
**Occupation of father** Health related Others	4 4	0.631	4 4	0.381
**Occupation of mother** Health related Others Housewife	4 3.5 4	0.376	4 4 4	0.622

Table 5 shows the suggested advantages of literature in medical education. A student stated, *“Medical Studies are often facts, facts and only facts. We lack situations where we can acquire knowledge critically. Thus this can help improve critical thinking skills”*. Respondents were asked whether they felt the use of English language excerpts in the module was appropriate. Fifty-one respondents (75%) felt they were appropriate. Among the reasons mentioned were English is the international language, the students were from English medium schools and were comfortable with English, the excerpts dealt with Nepal and English is the language of instruction. Eleven (16.2%) felt the excerpts were inappropriate. Cited reasons were they do not describe the Nepalese situation, Nepalese excerpts are available and English is not a commonly used language in Nepal. Forty-one respondents (60.3%) were easily able to understand the language used in the excerpts while 25 (36.8%) were not able to do so. Difficult words, tough language and low level of mastery of the English language were cited as reasons for difficulties.

**Table 5.** Advantages of literature in medical education.

**Suggestion**	**Number of respondents** **(percentage)**
Will prepare for future practice	13 (19.1)
Make them aware of various difficulties faced by doctors	12 (17.6)
This is a better and more interesting method of learning	9 (13.2)
Introduction to health issues in different parts of the world	7 (10.3)
Helps to understand feelings of patients	6 (8.8)
Can improve communication skills	5 (7.3)

The facilitators provided a brief introduction about the book and the author before distributing the excerpts. Fifty-eight respondents (85.3%) found this useful. The introduction often helped to put the excerpt in context, students knew more about the author and they could read the book if they were interested. Suggestions to further improve the use of literature excerpts were also obtained. Common among these were to also use Nepali language excerpts (16 respondents), use more excerpts (8), use excerpts with simpler language (6), use more excerpts from the medical field (5), provide photocopies of the excerpts before hand to the students (4), and provide more time for the activity (4 respondents).

Characteristics of students participating in the medical humanities module at KIST Medical College (Nepal) and their feedback scores on the use of literature excerpts within the module. Gender: 1 = male, 2 = female Financing: 1 = scholarship, 2 = self-financing Medium of instruction at school (medium): 1 = English, 2 = vernacular Place where their family is residing (residence): 1 = urban, 2 = rural Occupation of father (occufath): 1 = health science related, 2 = others Occupation of mother (occumoth): 1= health science related, 2 = others, 3 = housewife Enjoyment score (enjoy): score out of 5 Effectiveness score: score out of 5 The value 0 has been given where the scores were not available.

## Discussion

Participant feedback about the use of literature excerpts during the module was on the whole positive. They felt the excerpts used were not too difficult to understand and either reflected or could be extrapolated to the Nepalese scenario. They also wanted more scenarios by Nepalese authors and in the Nepal language to be used. They were strongly in favor of continuing the use of literature excerpts during future MH modules.

Most of the students are self-financing and only seven students were scholarship students. Scholarship students are selected on the basis of ranks obtained in an entrance examination conducted by the Ministry of Education, Government of Nepal. Compared to the self-financing students they are usually of lower socioeconomic background and from rural areas. These students have to serve for two years in rural areas after graduation and are becoming an important source of support to Nepal’s health system ^17^. KISTMC provides 10% of total seats on full tuition fees scholarship. In Nepal like in other parts of South Asia there are two media of instruction in schools. In vernacular medium schools all subjects are taught in the local language and English is taught as a second or third language. In English medium schools all subjects are taught in English and the native language is taught only as a second or third language. Most English medium schools are in the private sector and charge high tuition fees. Due to English being regarded as the international language and the language of science and education there is a high demand for English language education in the country. Most of our students were from English medium schools and from an urban background. They would have faced fewer problems in understanding and interpreting the English language excerpts. Medical education is expensive with most schools charging around 40,000 US dollars as tuition fees for the course.

The excerpts as stated were by foreign authors but were either about Nepal or dealt with health related topics. Students felt that these introduced them to different problems they may face in their future practice and also provided ideas about how to deal with these issues. Images of disease and death are common in literature and can serve to introduce students and doctors to experiences they may not have had themselves ^18^. Narrative methods are widely used in medicine with patients telling their stories to doctors, who tell these stories to other doctors during case presentations and the patient is regarded as a book needing interpretation ^19^. Study of literature can improve performance in the narrative aspects of medicine. We had used brief one-page excerpts in the module due to time and other constraints. Also literature was used as one of the modalities along with case scenarios, role-plays and paintings.

In California in the US a brief literature-based course was shown to contribute to greater student empathy and appreciation of the role of humanities in medical education ^20^. At the University of Oxford in the United Kingdom, a special study module using literature was shown to have increased insights gained into patients and their experience of illness and empathy. Certain clinically important skills were also increased ^21^. There have been many reviews highlighting the importance of MH and literature and its perceived advantages in undergraduate medical education. A recent review however, concludes that there is a shortage of studies describing long-term impact of MH on the development of medical proficiency ^22^. The authors conclude that this may pose a threat to the continued development of MH as administrators and curriculum planners may demand objective evidence of its effectiveness. In Nepal at present to the best of our knowledge we are the only medical school offering a MH module and the first batch of students are now in their fourth year of study. Studying effectiveness in terms of professional behaviors will be important for continued growth of the discipline.

The books and the excerpts used have been already described. The excerpts were in English and most of the students were comfortable with the English language due to reasons previously mentioned. Two of the authors of this article (PRS and RMP) had previously explored the issue of English as the language of MH teaching ^23^. In South Asia due to a variety of reasons among them the British colonial influence, English is the language of higher education and the medium of instruction in medical schools. Though Nepal was not ruled by a colonial power, it was strongly influenced by India especially in the field of education. English as the language of excerpts has both advantages and disadvantages. In many developing countries there are a number of languages spoken and it may be difficult to find a language acceptable to everyone ^23^. In KISTMC nearly all our students are from Nepal and it may be possible to use Nepali language excerpts. This was not possible at MCOMS, Pokhara where there were students of different nationalities ^4^. However, English language excerpts are easier to find and use compared to native language ones. In our previous modules at MCOMS ^4^ and the faculty module at KISTMC ^5^ participants had difficulty with the level of English in certain excerpts. These complaints were fewer this time as we had taken care to select excerpts with simpler language.

A few students had volunteered to provide Nepali language excerpts and we can consider this during future modules. The excerpts were short and certain participants had problems putting the excerpt in context. Opinion is divided among facilitators and others about whether to select the majority of excerpts from medicine and related areas. Some feel that this is important as students will be practicing doctors once they qualify while others feel medicine, illness and death are a part of life and cannot be divorced from the larger scenario. Providing photocopies of the excerpts one day before to all students can be considered.

A recent article has stated that MH could be considered as representative of western culture ^24^. The authors state that MH programs in Asia promoted a quasi-Western approach to medical humanities (in history, philosophy, literature and art) and the diversity, sophistication and richness of different cultural traditions was uncomfortably marginalized. The authors further state that MH curricula concentrated on two genres of work. These were works in literature, arts and music valued for their insight and beauty and ‘experiential’ works written by both doctors and patients. The issue of indigenous cultural traditions being marginalized requires attention. In South Asia English is increasingly dominant as the language of education and culture. Western cultural values are strongly promoted through the mass media and the internet. How to involve more indigenous cultural expressions in MH modules can be a major challenge.

Our experiences with the use of literature excerpts would be of interest to medical educators planning to introduce MH modules in other developing nations. At present these modules are few in number but many medical schools are interested in introducing a certain amount of MH teaching in the curriculum. In many developing nations the medium of instruction is either English or other colonial languages. Also in most developing nations only students from a science background are eligible to study medicine. In South Asia twelve years of schooling with the subjects of Physics, Chemistry and Biology during the last two years is necessary. Medical students are less exposed to literature and the humanities and sometimes have a negative perception of these disciplines.

The strength of the study is the high response rate. Our study had limitations. Student perception regarding the use of literature excerpts was only studied using a questionnaire. Other methods were not used. The questions included were arrived at by consensus among the authors. The questionnaire was pretested for comprehension and understanding among five third year students. We however felt that a few respondents had difficulty in properly comprehending certain questions. There is a certain amount of repetition in the responses obtained.

## Conclusions

The use of literature excerpts was appreciated by the students. Most felt English language excerpts were acceptable but felt Nepali language excerpts could also be considered. Students strongly felt that the use of literature excerpts should be continued in future MH modules. This use will provide us with more information regarding student understanding and acceptance of the same. The type of excerpts to be used and its length have to be decided based on consensus among facilitators. Our experiences would be of interest to educators planning to introduce MH modules in other developing nations.
