# Supplementary material for: Student feedback about the use of literature excerpts in Sparshanam, a Medical Humanities module
Source: F1000Res. 2012 Nov 15;1:49. [Version 1] doi: 10.12688/f1000research.1-49.v1 (PMC3752629; doi:10.12688/f1000research.1-49.v1)
Supplement: Student feedback questionnaire — about the use of literature excerpts in Sparshanam, the KIST Medical College (Nepal) medical humanities module [file f1000research-1-225-s0001.tgz › Student_feedback_about_the_use_of_literature_excerpts_in_Sparshanam.pdf]

Student feedback about the use of literature excerpts in Sparshanam, the KISTMC Medical Humanities module

Gender: M/F

Self-financing/Scholarship

Medium of instruction at school: English/Nepali/Others (specify)

Place of family residence: Urban/Rural

Occupation of parents: Father: Mother:

Give TWO overall comments about the use of literature excerpts in Sparshanam:

Have you exposed to the use of literature excerpts for educational purposes before? If yes, give details.

According to you how did literature excerpts help in realizing the objectives of the module? (Two points)

Are you aware of the use of literature excerpts in medical education elsewhere? If yes, give mention them.

Grade your enjoyment of literature excerpts used in the module on a on a scale of 1 to 5 with 1 being least and 5 being most enjoyable.

Do you feel use of literature excerpts from a predominantly western context were appropriate in the module?
Give your reasons.

Which of the various excerpts used could you identify with the most? Why?

Which of the various excerpts used could you identify with the least? Why?
What would you suggest to make this exercise more useful?

Did you have any difficulty in putting the scenarios depicted in the literature excerpts in a Nepalese context?

If yes, then how did you overcome these?

Do you feel literature excerpts in Nepali and by Nepali authors should be used in the module?

If yes, then how should we go ahead?

Do you feel literature excerpts should be used in future modules?

Mention TWO advantages of literature for medical students.

Do you feel literature excerpts in English are appropriate in a Nepalese context? Why?

Were you able to easily understand the language used in the literature excerpts?

Do you feel the brief introduction to the book and the author provided before the literature excerpts were useful?

How would you rate the effectiveness of literature excerpts used in the module on a scale of 1 to 5 (with 1 being least and 5 being most useful)

Give TWO suggestions to further improve the use of literature excerpts in the future

Thank you for completing the questionnaire. It is very much appreciated!
