# Supplementary material for: Student feedback about the use of literature excerpts in Sparshanam, a Medical Humanities module
Source: F1000Res. 2012 Nov 15;1:49. [Version 1] doi: 10.12688/f1000research.1-49.v1 (PMC3752629; doi:10.12688/f1000research.1-49.v1)
Supplement: Peer review history

# Peer review: Referee response for version 1

**Published:** 2012-12-17

**Authors:** Avinash Supe

**Affiliations:** Gordhandas Sunderdas Medical College, King Edward Memorial Hospital, Mumbai, India

I have read this submission. I believe that I have an appropriate level of expertise to confirm that it is of an acceptable scientific standard.

# Peer review: Referee response for version 1

**Published:** 2012-12-17

**Authors:** Amol Dongre

**Affiliations:** Department of Community Medicine, Sri Manakula Vinayagar Medical College and Hospital, Pondicherry, India

I have read this submission. I believe that I have an appropriate level of expertise to confirm that it is of an acceptable scientific standard.

# Peer review: Referee response for version 1

**Published:** 2012-11-22

**Authors:** Carol Ann Courneya

**Affiliations:** Department of Cellular and Physiological Sciences, University of British Columbia, Vancouver, Canada

I have read this submission. I believe that I have an appropriate level of expertise to confirm that it is of an acceptable scientific standard.
